# Resurfacing Hip Arthroplasty Is a Safe and Effective Alternative to Total Hip Arthroplasty in Young Patients: A Systematic Review and Meta-Analysis

**DOI:** 10.3390/jcm12062093

**Published:** 2023-03-07

**Authors:** Michele Palazzuolo, Alessandro Bensa, Stefan Bauer, William G. Blakeney, Giuseppe Filardo, Martin Riegger

**Affiliations:** 1 Service of Orthopaedics and Traumatology, Department of Surgery, EOC, 6600 Locarno, Switzerland; 2 Service of Orthopaedics and Traumatology, Department of Surgery, EOC, 6900 Lugano, Switzerland; 3 Service d’Orthopédie et de Traumatologie, Chirurgie de l’Épaule, Ensemble Hospitalier de la Côte, 1110 Morges, Switzerland; 4 Department of Orthopedic Surgery, Royal Perth Hospital, Perth, WA 6000, Australia; 5 Faculty of Biomedical Sciences, Università della Svizzera Italiana, 6900 Lugano, Switzerland

**Keywords:** hip, resurfacing arthroplasty, total arthroplasty, RHA, THA, ion level

## Abstract

This systematic review and meta-analysis of randomized controlled trials (RCTs) aimed at comparing resurfacing hip arthroplasty (RHA) and total hip arthroplasty (THA) in terms of rate of complications, revisions, functional outcomes, blood loss, operative time and metal ions levels. The search was conducted on three databases (PubMed, Cochrane and Web of Science) updated until 13 October 2022. The inclusion criteria were RCTs) written in the English language, with no time limitation, comparing RHA and THA. Among the retrieved 4748 articles, 18 RCTs were eligible for a total of 776 patients (mean age 53.1 ± 5.0). A meta-analysis was performed. RHA reported significantly lower blood loss compared to THA (*p* < 0.001) but with longer operative time (*p* < 0.001). No statistically significant difference was found between RHA and THA in terms of complications (12.08% and 16.24%, respectively) and revisions (6.32% and 6.14%, respectively). Both RHA and THA provide excellent clinical results in a population of young and active patients. Functional outcomes were not significantly different between the groups. Moreover, no significant difference in metal ion levels was found. These findings provide evidence concerning the safety and clinical effectiveness of RHA. Because of its bone-preserving properties, the lack of drawbacks and good outcomes, RHA appears to be a valid alternative to THA in young and active patients.

## 1. Introduction

**Figure 1:**
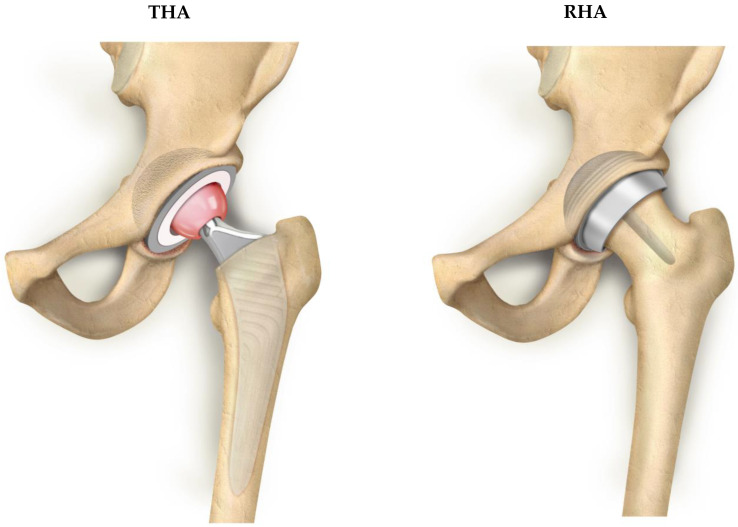
Illustration of total hip arthroplasty (THA) and resurfacing hip arthroplasty (RHA).

Total hip arthroplasty (THA) is the mainstay of treatment for patients with end-stage hip osteoarthritis. It is largely performed worldwide and demonstrates a proven record of success [1]. In the last two decades, resurfacing hip arthroplasty (RHA) has been developed as a potential alternative to THA in selected subgroups of young and active patients, aiming for higher femoral bone stock preservation (Figure 1) [2,3,4]. In the late 1990s and early 2000s, the lack of knowledge about RHA design, tribology and mechanical properties had led to much interest but also to this procedure being abandoned. In this respect, the biggest downsides of this procedure are that it cannot be performed in all hip cases and that it has a rather long learning curve [5]. However, several surgeons worldwide are reconsidering the RHA technique benefiting from a more anatomical treatment approach. Registries in several countries report favorable results for RHA [6], leading to a renewed interest in this procedure and to RHA gaining ground over THA with certain designs and in selected patient subgroups [7]. Apart from minimal bone resection, other theoretical advantages of RHA include improved joint stability, better reproduction of native hip biomechanics and diminished linear wear. Some studies have also suggested a functional advantage for hip resurfacing suggesting even higher function scores in patients who had undergone RHA instead of THA [7]. However, concerns with metal ions that are potentially generated in RHA have led to a dramatic reduction in the use of hip resurfacing and to the withdrawal of some implants [8,9,10].

Despite the existing literature with several articles comparing RHA and THA, current evidence provides studies with conflicting results. A comprehensive analysis quantifying the pros and cons of the resurfacing approach versus the more classic THA would be beneficial to help physicians in the choice of the most suitable treatment approach.

The aim of this systematic review and meta-analysis was to compare RHA and THA in terms of rate of complications, revisions, functional outcomes, blood loss, operative time and metal ions levels to provide indications on the most suitable procedure to address patients affected by hip osteoarthritis.

## 2. Methods

### 2.1. Search Strategy

This systematic review and meta-analysis was performed according to the Preferred Reporting Items for Systematic Reviews and Meta-Analysis (PRISMA) [11,12]. A comprehensive literature search was conducted on the PubMed, Cochrane and Web of Science databases on 13 October 2022 using the following search string: (hip) AND (prosthe* OR arthroplasty OR tha OR total hip OR replacement OR THR) AND (HRS OR RHA OR resurfacing).

### 2.2. Study Selection

Two reviewers (M.P. and A.B.) were involved independently in the screening and extraction process, with disagreements resolved by consensus with a third author (M.R.). First, the articles were screened by title and abstract. The following inclusion criteria were used: randomized controlled trials (RCTs), written in the English language, with no time limitation and on the comparison of THA and RHA. Exclusion criteria were articles written in other languages, reviews, preclinical studies, non-randomized trials and studies not reporting clinical outcomes or laboratory parameters. The studies were first manually screened based on the title and abstract. In the second step, the full texts of the selected articles were screened, with further exclusions according to the previously described criteria.

### 2.3. Data Extraction

Three reviewers (A.B., M.P. and M.R.) independently extracted the data according to a standardized form. For each selected article, the following information was collected: title, first author, publication year, country, participant characteristics, study design, sample size, follow-up, complications, revisions, operative time, blood loss, blood cobalt and chromium levels, and functional scores (WOMAC, UCLA and HHS), expressed as mean or median, with either standard deviation (SD), standard error (SE) or confidence interval (CI), or Inter Quartile Range (IQR). Any discrepancy was solved through discussion. After tabulation, a merging of the extracted data was performed. For studies with insufficient information, more information was searched on the ClinicalTrials.gov platform or by contacting the corresponding author.

### 2.4. Quality Assessment

The quality of each article was assessed using the Cochrane risk-of-bias tool for randomized trials Version 2 (RoB 2). RoB 2 is structured into a fixed set of domains of bias, focusing on different aspects of trial design, conduct and reporting. Within each domain, a series of questions (‘signaling questions’) aim to elicit information about features of the trial that are relevant to the risk of bias. A proposed judgment about the risk of bias arising from each domain is generated by an algorithm based on answers to the signaling questions. Judgment can be ‘Low’ or ‘High’ risk of bias or can express ‘Some concerns’ [13].

### 2.5. Statistical Analysis

Statistical analysis and Forest plotting were carried out according to Neyeloff et al. [14] using the Meta XL tool for Microsoft Excel. The analysis was carried out using random effects (DerSimonian and Laird) for the weighted mean difference of the continuous variables and the Peto method for odds ratios of the dichotomous variables. The I-Square statistic for heterogeneity was included, as well as the Q statistic. In the case of the continuous outcome, the weighted mean difference (delta) was used to calculate the Z test statistic. The confidence intervals (95% CI) for delta were then derived, and if the 95% CI excludes zero, then evidence exists that the meta-analysis of interest has shown a significant treatment effect at 0.05 level of significance. In addition, the derived results were used to define the test statistic z = delta/SE, which is N(0, 1). Therefore its corresponding P value can be used to confirm or negate the reported result of the same meta-analysis. For the dichotomous variables, similarly, the odds ratio (OR) was used to calculate the test statistic. The confidence interval (95% CI) for OR was then derived, and if the 95% CI excludes zero, then evidence exists that the meta-analysis of interest has shown a significant treatment effect at 0.05 level of significance; the Fisher exact test was then used to check if the odds ratio was statistically different from 1.

## 3. Results

### 3.1. Literature Search

**Figure 2:**
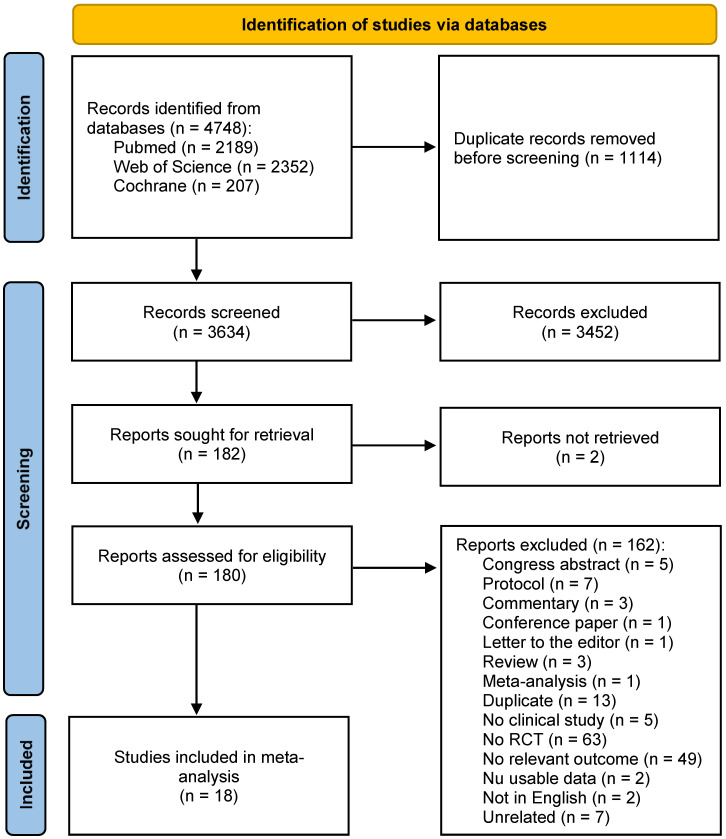
Study selection flow chart.

The literature search results are summarized in Figure 2 and briefly described below.

**Figure 3:**
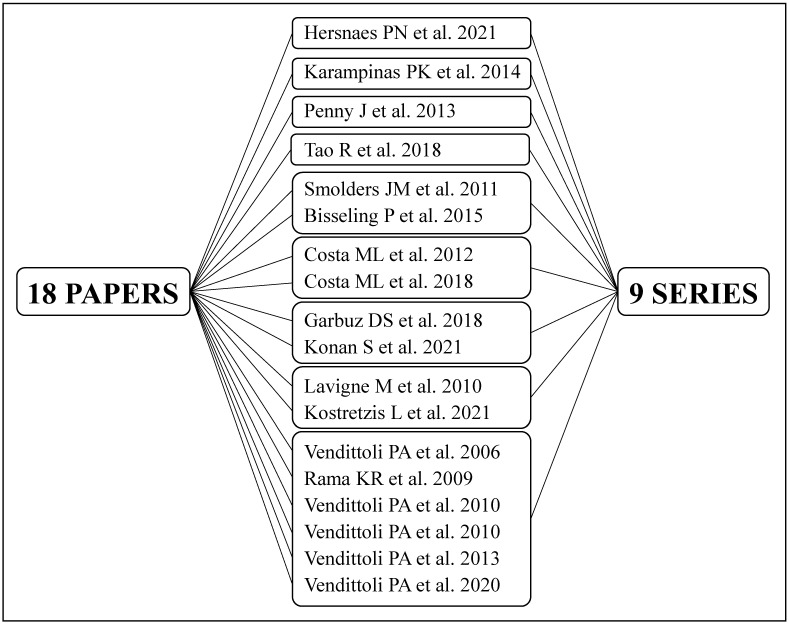
Correspondence between the 18 articles retrieved and the 9 series of patients treated [15,16,17,18,19,20,21,22,23,24,25,26,27,28,29,30,31,32].

A total of 4748 articles were retrieved; after the removal of duplicates, and screening of the titles, abstracts and full texts, 18 RCTs were included according to the eligibility criteria. Characteristics and technical aspects of the 18 eligible studies are shown in Table 1. Among the 18 studies included, 9 were found to be follow-ups of previous publications and therefore referring to the same original series of patients: 9 series of patients were therefore identified, and the most complete data extrapolated from the relative studies were included in the qualitative analysis (systematic review) and quantitative analysis (meta-analysis), as depicted in Figure 3 [15,16,17,18,19,20,21,22].

### 3.2. Qualitative Analysis

#### 3.2.1. Study Characteristics

A total of 776 patients (67.1% males and 32.9% females, mean age 53.1 ± 5.0) were analyzed: 373 patients (65.4% males and 34.6% females, mean age 52.5 ± 5.3) in the RHA group and 403 patients (68.7% males and 31.3% females, mean age 53.6 ± 4.9) in the THA group. Different clinical scores were used to evaluate patients, the most used being Western Ontario and McMaster Universities Osteoarthritis Index (WOMAC, five series of patients), University of California at Los Angeles (UCLA) score (seven series) and Harris Hip Score (HHS, six series). Complications and revisions were reported in eight patient series, while operative time and intraoperative blood loss were reported in four and three series, respectively. Cobalt and chromium levels were reported in five patient series. The mean follow-up was 7.2 ± 4.7 years.

#### 3.2.2. Main Findings

The main findings of the 9 patient series in the 18 RCTs included are depicted in Table 2. No study found any statistically significant difference in terms of complication and revision rates between RHA and THA. Only two papers suggested two opposing trends of revision rates: Konan et al. [23] reported better results for the RHA group, while Hersnaes et al. [24] presented better results for the THA group, but none of the two found a statistically significant difference. Moreover, it is relevant to underline that the study published by Hersnaes et al. was prematurely terminated due to numerous reports of adverse events in patients who underwent metal-on-metal hip replacements.

Peri-operative parameters showed a tendency to favor THA over RHA in some studies. In fact, significantly lower operative time was reported in four series of patients [25,26,27,28] and significantly shorter incision length in two series of patients [25,29] in favor of THA. No paper found a statistically significant difference in terms of blood loss, even if a trend of lower values in RHA was reported in three series of patients [25,26,28].

The two procedures were not found to be statistically different in terms of functional outcomes by most of the studies. Only Kostretzis et al. [30] found significantly better WOMAC values in THA patients compared to RHA patients at a mean follow-up of 14 years. On the other hand, Bisseling et al. [27] demonstrated significantly superior UCLA and HHS values in RHA compared to THA at 6 months of follow-up and better UCLA values at 1 year follow-up. In the series of patients analyzed by Vendittoli et al. [29], significantly better UCLA values were reported in RHA patients at a mean follow-up of 8 years, but this difference was lost at 15 years of follow-up [28].

The results regarding blood ion levels were heterogeneous, with two series of patients [23,30] reporting significantly lower cobalt and chromium values in patients who underwent RHA and other another series of patients [27] demonstrating lower values in THA patients. Moreover, the series of patients analyzed by Vendittoli et al. [31] showed significantly lower cobalt and chromium values at 3 months of follow-up, but this difference disappeared from the 2-years follow-up, while a significant difference in terms of blood titanium values was reported up to the 5-years follow-up in favor of THA.

### 3.3. Meta-Analysis

#### 3.3.1. Complications and Revisions

##### Complications

**Figure 4:**
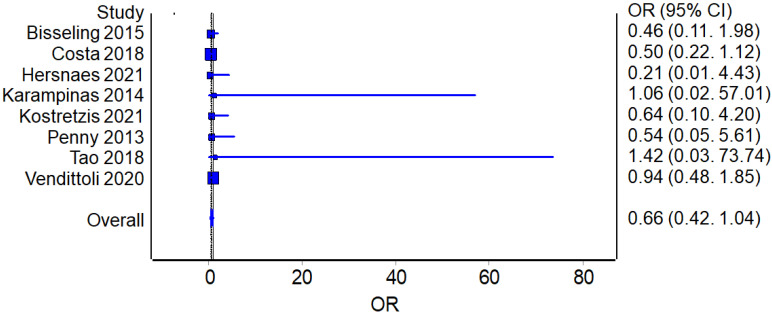
Complications Rates: Forest plot of the individual studies and pooled mean difference for complication rates, including a 95% confidence interval. The size of the squares shows the weight of the study [15,16,17,18,19,20,21,22,24,25,26,27,28,29,30,31].

Eight studies reported the number of complications for the RHA (325 patients in total) and THA procedures (347 patients in total). The overall odds ratio (OR) analysis found a value of 0.66 in favor of RHA, although without reaching a statistically significant difference between the two groups (*p* = 0.071) (Figure 4).

##### Revisions

**Figure 5:**
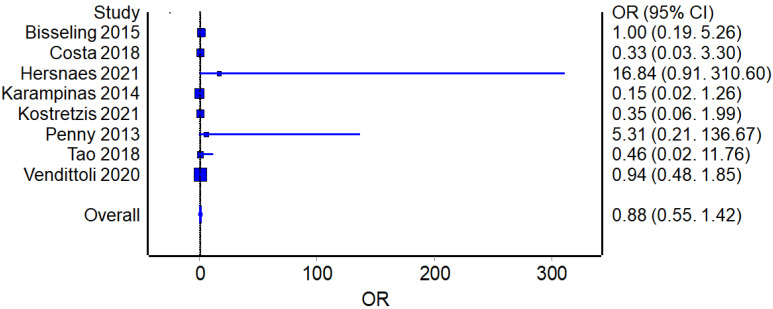
Revision Rates: Forest plot of the individual studies and pooled mean difference for revision rates, including a 95% confidence interval. The size of the squares shows the weight of the study [15,16,17,18,19,20,21,22,24,25,26,27,28,29,30,31].

Eight studies reported the number of revisions for the RHA (358 patients in total) and THA procedures (387 patients in total). The overall OR analysis found no statistically significant difference between the two groups (Figure 5).

#### 3.3.2. Perioperative Parameters

##### Operative Time

**Figure 6:**
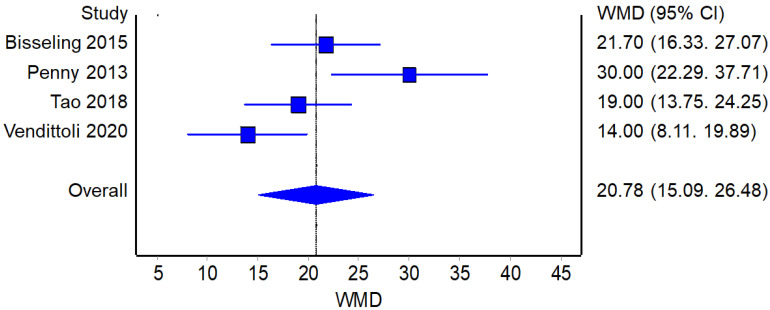
Operative Time: Forest plot of the individual studies and pooled mean difference for operative time, including a 95% confidence interval. The size of the squares shows the weight of the study [17,18,19,21,25,26,27,28,29,31].

Four studies reported the operative time for the RHA (190 patients in total) and THA procedures (206 patients in total). A statistically significant difference was found between the two groups (*p* < 0.001), with RHA demonstrating a longer operative time compared to THA (Figure 6).

##### Blood Loss

**Figure 7:**
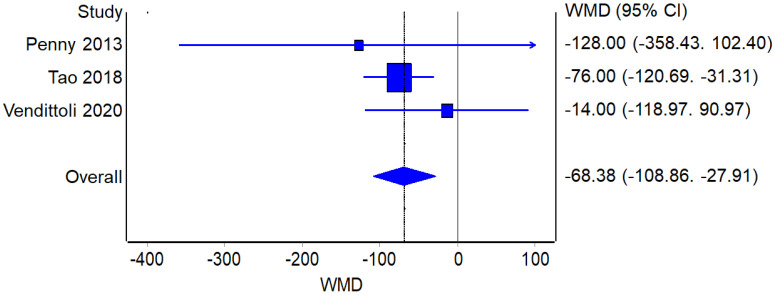
Blood Loss: Forest plot of the individual studies and pooled mean difference for blood loss, including a 95% confidence interval. The size of the squares shows the weight of the study [17,18,19,25,26,28,29,31].

Three studies reported blood loss for the RHA (152 patients in total) and THA procedures (173 patients in total). A statistically significant difference was found between the two interventions (*p* < 0.001), with RHA demonstrating lower blood loss compared to THA (Figure 7).

#### 3.3.3. Functional Outcomes

##### WOMAC

**Figure 8:**
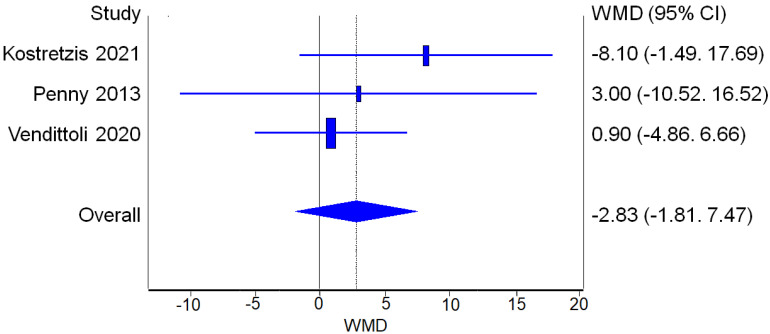
WOMAC: Forest plot of the individual studies and pooled mean difference for WOMAC improvement, including a 95% confidence interval. The size of the squares shows the weight of the study [17,18,19,20,25,28,29,30,31].

Three studies reported WOMAC score values before and after the surgical procedure for both RHA and THA. The analysis of WOMAC improvement from the pre-op values to the post-op values did not find a statistically significant difference between the two procedures (Figure 8).

##### UCLA

**Figure 9:**
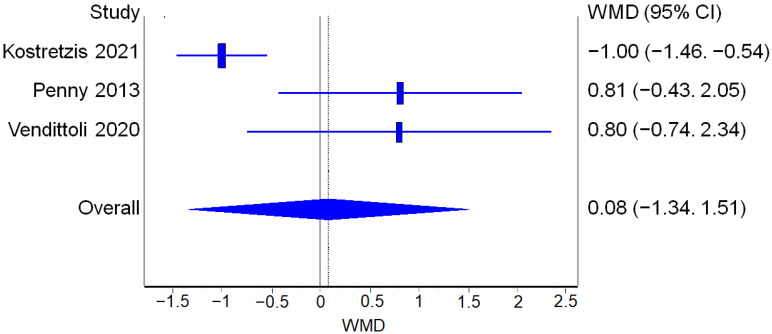
UCLA: Forest plot of the individual studies and pooled mean difference for UCLA improvement, including a 95% confidence interval. The size of the squares shows the weight of the study [17,18,19,20,25,28,29,30,31].

Three studies reported UCLA score values before and after the surgical procedure for both RHA and THA. The analysis of UCLA improvement from the pre-op values to the post-op values did not find a statistically significant difference between the two procedures (Figure 9).

##### HHS

**Figure 10:**
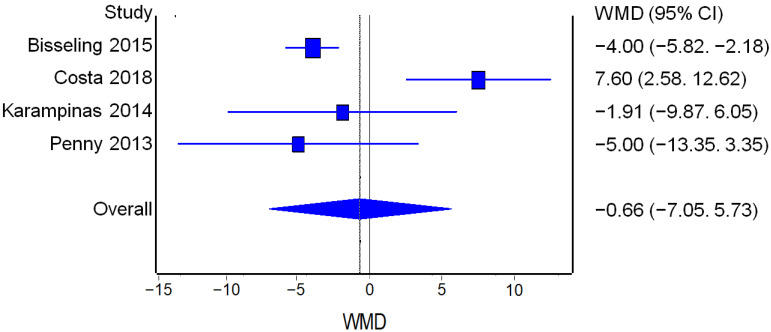
HHS: Forest plot of the individual studies and pooled mean difference for HHS improvement, including a 95% confidence interval. The size of the squares shows the weight of the study [15,16,21,22,25,27].

Four studies reported HHS score values before and after the surgical procedure for both RHA and THA. The analysis of HHS improvement from the pre-op values to the post-op values did not find a statistically significant difference between the two procedures (Figure 10).

#### 3.3.4. Metal Ions Levels

Post-operative blood cobalt and chromium levels were reported in three studies for both RHA (121 patients in total) and THA procedures (113 patients in total).

##### Cobalt Levels

**Figure 11:**
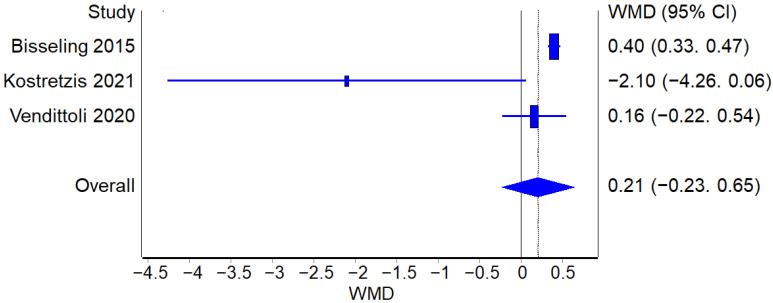
Cobalt Levels: Forest plot of the individual studies and pooled mean difference for blood cobalt levels, including a 95% confidence interval. The size of the squares shows the weight of the study [17,18,19,21,27,28,29,30,31].

The analysis of blood cobalt levels after the intervention did not show a statistically significant difference between RHA and THA procedures (Figure 11).

##### Chromium Levels

**Figure 12:**
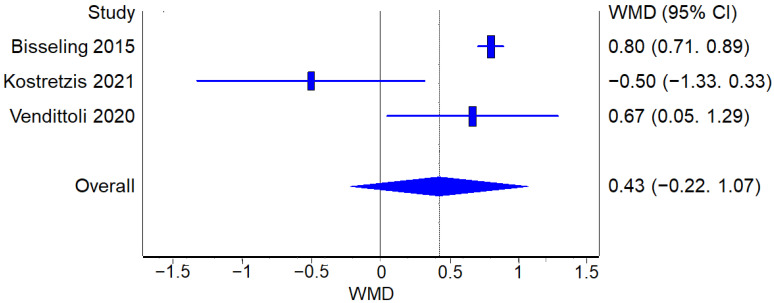
Chromium Levels: Forest plot of the individual studies and pooled mean difference for blood chromium levels, including a 95% confidence interval. The size of the squares shows the weight of the study [17,18,19,21,27,28,29,30,31].

The analysis of blood chromium levels after the intervention did not show a statistically significant difference between RHA and THA procedures (Figure 12).

### 3.4. Quality Assessment

The evaluation using the RoB2 tool showed an overall heterogeneous quality of the studies, with 3 papers falling in the “High risk” category, 3 in the “Some concerns” category and 12 in the “Low risk” category. Detailed results are shown in Table 3.

## 4. Discussion

The main findings of this study are that RHA is a suitable alternative to THA. In the investigated population of young to middle-aged patients, while RHA presented a longer operative time, it also caused significantly lower blood loss and similar satisfactory clinical results, with no significant difference in metal ion levels.

These findings shed new light on the interpretation of the controversial literature results questioning the benefits and risks of RHA. This meta-analysis supports the use of RHA, as previously suggested, for a selected population of young and athletic patients [7]. Theoretical advantages of RHA over THA include a return to high-level sports [33,34], low rates of linear wear, preservation of femoral bone stock [35], potentially improved coxo-femoral kinematics [36], and low risk of instability and dislocation [37]. In spite of these many theoretical advantages, with the excellent implant survivorship and functional outcomes seen in THA, it is still unclear whether RHA actually provides a clinically relevant benefit over THA [38]. Potential advantages of RHA must also be balanced against the possible disadvantages related to the metal-on-metal bearing couple, including an abnormal increase in blood cobalt and chromium ion levels [39]. Due to the small sample size and heterogeneity of the existing studies, adequate interpretation of the current evidence requires a broad literature investigation to quantify the claimed drawbacks. With this systematic review and meta-analysis, we aimed to compare RHA and THA in terms of the overall rate of complications and revisions, functional outcomes, peri-operative parameters and metal ions levels.

Overall, the present meta-analysis of RCTs did not demonstrate a statistically significant difference in the rate of complications between RHA and THA. Despite not reaching the level of statistical significance, even a trend in favor of RHA was noted with a tendency towards a lower complication rate in RHA. Moreover, no significant difference in the revision rate was apparent between the two groups. These findings provide evidence concerning the safety of the RHA procedure, which is not affected by a higher complication or revision rates compared to THA. In another meta-analysis by Hellmann et al. [38], fracture and infection rates were similar between RHA and THA, while dislocation rates were lower in RHA compared with THA. The efficacy and safety of RHA translate in an implant survival comparable to THA, with the current meta-analysis documenting 23 and 24 revisions, respectively. Other studies compared RHA to THA survivorship. Palazzuolo et al. investigated 427 patients (286 THA and 141 RHA) and reported survivorship at 10 years of 89% and 96% for THA and RHA, respectively [40]. Lons et al. investigated 481 patients and reported survivorship at 4 years of 99.4.% for RHA [2]. However, a recent study on registry data suggests that THA with proven low revision rates might be a better choice considering the concerns about implant durability and metal ions levels [41]. In this regard, studies with long-term survival rates (>20 years) for RHA are still lacking, and because of the impact of metal ion production in RHA over the years, further evidence is needed to compare the long-term survivorship of the two types of implants.

The present systematic review and meta-analysis did not find any difference in blood cobalt and chromium ion levels between the two groups. Some studies highlighted a higher proportion of adverse local tissue reactions (ALTRs) or metallosis on MRI in patients with RHA compared with patients with THA, even if patient self-assessed symptoms were not different between patients with ALTR or metallosis on MRI and patients with absence of these features [42]. Despite the concern for ALTRs and metallosis, metal ions production in RHA has been shown to be far inferior compared to large head metal-on-metal THA, as most of the ions production comes from the trunnion and, by definition, RHA is lacking the head-neck junction [40]. In this regard, because of similar function and revision rates, some authors concluded that ionic levels might not be a detrimental issue as they do not seem to negatively impact implant function and survivorship [43]. However, it is relevant to note that RHA survivorship varies significantly according to the different existing implants, possibly because of their different alloys and mechanical properties. As an example, the Articular Surface Replacement RHA (ASR; DePuy, Leeds, UK) and the Durom RHA (Zimmer, Warsaw, IN) have been recalled from the market due to a high prevalence of ALTRs and a high early revision rate [44]. On the other hand, the survival rate of the Birmingham Hip Resurfacing (Smith and Nephew, Warwick, UK) has been higher than that of all other RHA devices and has demonstrated excellent survivorship [2,45]. As such, proper implant selection appears to be a major determinant of implant survivorship and revision rate [4].

The present study did not find any significant difference in terms of functional outcomes between RHA and THA: in fact, no statistically significant differences emerged between the two groups in terms of WOMAC score, UCLA score or HHS. These findings reflect the existing literature, as the post-operative functional outcome was good to excellent for both RHA and THA in most of the existing series. In the study by Garbuz et al., both groups reported an improvement in quality of life and activity scores, but no difference was found between the two groups [32]. The same applies to the findings of Costa et al., who reported no difference in hip function between the treatment groups at 12 months [15]. In the meta-analysis by Hellmann et al., RHA demonstrated equivalent patient-reported outcome scores with greater activity scores and a return to high-level activities compared with THA [38]. Some other studies even demonstrated a superior functional outcome for RHA, as in the recent meta-analysis by Kumar et al., where HHS was found to be significantly better in the RHA group [43]. Moreover, in the studies that showed no significant difference between RHA and THA, no activity-specific measures were used. Evidence exists that RHA may offer some potential advantages over THA in this sense, including an early return to high-level activities and sports [46], restoration of native hip biomechanics and decreased proximal femoral stress shielding [38]. It appears that the potential advantages of RHA could be shown only in the studies that used physical activity-specific outcome measures, as was suggested in the existing literature analyzing the return to sport after RHA [33,38,47,48,49]. As such, well-controlled prospective studies focusing on clinically important differences in patient-reported outcomes and functional results comparing RHA and THA prostheses with modern bearings are still needed.

In the present series, a statistically significant difference between the two groups was found in terms of intraoperative blood loss and surgical time. RHA procedures were significantly longer than THA. The longer operative time could probably be explained by the complexity of the RHA procedure that requires perfect component positioning. Perfect implant positioning in RHA is critical in order to avoid femoral neck fracture (in case of varus positioning of the femoral component) and edge loading (too-vertical acetabular cup positioning), which can cause uneven force distribution at the metal–metal interface, thereby drastically increasing metal ion production [50]. Despite the longer operative time, RHA procedures were associated with significantly lower intraoperative blood loss. While this may seem counterintuitive, the lower blood loss could probably be explained by the fact that, by definition, the neck of the femur is not cut during RHA procedures, thus avoiding a significant source of intraoperative bleeding.

This study presents some limitations. Despite a mean follow-up of more than seven years, some of the studies included in the meta-analysis had a relatively short follow-up. As such, future studies should confirm these findings at longer follow-ups. The examined RCTs did not include any physical activity-specific outcome measures, relying on general function scores (WOMAC, UCLA, HHS). Even though the WOMAC Index is self-administered and assesses the three dimensions of pain, disability and joint stiffness in knee and hip osteoarthritis using a battery of 24 questions, with proven validity in orthopedic outcome studies for the assessment of the effectiveness of surgery such as THA [51], its ability to detect a change in functional status is limited due to the overlap of pain and function items [52]. Another limitation is that RHA and THA implants analyzed were not the same in all the studies. This may be particularly problematic for THA, as the bearing couple varied across the different studies examined and included both large-head metal-on-metal THA and conventional ceramic-on-polyethylene implants, thus potentially limiting the strength of our findings. However, this limitation was intrinsic to the nature and heterogeneity of the existing RCTs. Analogously, different RHA implants were pooled for the purpose of this study, while different products may be more prone than others to complications and metal ions levels. In order to solve this issue, well-conducted prospective studies comparing solely selected RHA and conventional ceramic-on-polyethylene implants in terms of activity-specific functional outcome measures should be carried out. Despite these limitations, this RCT meta-analysis offers important elements contributing to the scientific discussion on this topic and helping surgeons in the choice of the most appropriate management of young, middle-aged patients requiring hip replacement surgery.

## 5. Conclusions

Both RHA and THA provide satisfactory results in young and middle-aged patients. While RHA presented a longer operative time, it also caused significantly lower blood loss and similar satisfactory functional results, with no significant difference in metal ion levels. Complication and revision rates were also not significantly different between the groups. These findings provide evidence concerning the safety and clinical effectiveness of RHA.

## Figures and Tables

**Table 1:** Characteristics and technical aspects of eligible studies.

Author	Year	Journal	Study Type	Treatment Group	N° pts	M	W	Age	BMI
Bisseling P et al. [27]	2015	The Journal of arthroplasty	RCT	RHA	42	21	17	57.5	26.1
				THA	42	21	12	59.2	28
Costa ML et al. [22]	2018	BMJ open	RCT	RHA	60	36	24	56.5	28.4
				THA	62	35	27	56.7	28.9
Costa ML et al. [15]	2012	BMJ (Clinical research ed)	RCT	RHA	60	38	22	56.3	28.6
				THA	66	36	30	56.6	28.7
Garbuz DS et al. [32]	2010	Clinical orthopaedics and related research	RCT	RHA	48	43	5	51.5	28.3
				THA	56	50	6	52	28.2
Hersnaes PN et al. [24]	2021	BMC musculoskeletal disorders	RCT	RHA	36	26	10	59.4	27.45
				THA	39	26	13	61.9	28.4
Karampinas PK et al. [16]	2014	Orthopedic reviews	RCT	RHA	20	7	8	50.5	31
				THA	21	11	5	50.7	31.6
Konan S et al. [23]	2021	Hip international: the journal of clinical and experimental research on hip pathology and therapy	RCT	RHA	48	43	5	51.5	28.3
				THA	56	50	6	52	28.2
Kostretzis L et al. [30]	2021	BMC musculoskeletal disorders	RCT	RHA	24	14	10	50	28
				THA	24	15	9	50	28
Lavigne M et al. [20]	2010	Clinical orthopaedics and related research	RCT	RHA	24	14	10	49.6	27.9
				THA	24	15	9	49.8	27.8
Penny J et al. [25]	2013	Acta orthopaedica	RCT	RHA	20	12	8	57	28
				THA	34	24	10	56	27
Rama KR et al. [17]	2009	The Journal of Arthroplasty	RCT	RHA	109	65	38	50	27.3
				THA	100	66	31	50.3	29.7
Smolders JM et al. [21]	2011	Acta orthopaedica	RCT	RHA	42	21	17	58	26
				THA	42	21	12	59	28
Tao R et al. [26]	2018	International orthopaedics	RCT	RHA	28	19	9	43	21.5
				THA	40	28	12	47	21.8
Vendittoli PA et al. [31]	2006	Hip international: the journal of clinical and experimental research on hip pathology and therapy	RCT	RHA	109	67	40	49.1	27.2
				THA	100	70	33	50.6	29.6
Vendittoli PA et al. [18]	2010	Hip international: the journal of clinical and experimental research on hip pathology and therapy	RCT	RHA	109	69	40	49.2	27
				THA	100	68	32	51	30
Vendittoli PA et al. [19]	2010	The Journal of bone and joint surgery. British volume	RCT	RHA	109	42	22	49.3	27.1
				THA	100	33	20	51	29.2
Vendittoli PA et al. [29]	2013	Bone & Joint Journal	RCT	RHA	109	66	38	49.2	27
				THA	100	67	32	51	30
Vendittoli PA et al. [28]	2020	Journal of Bone and Joint Surgery-American Volume	RCT	RHA	109	66	38	48.9	26.6
				THA	100	67	32	50.7	30

**Table 2:** Main findings of the 9 patient series included.

Series of Patients	Treatment Type	Complications	Revisions	Operative Time (min)	Blood Loss (ml)	Incision Lenght (cm)	WOMAC Pre-op	WOMAC Post-op	UCLA Pre-op	UCLA Post-op	HHS Pre-op	HHS Post-op	Cobalt Level Pre-op	Cobalt Level Post-op	Chromium Level Pre-op	Chromium Level Post-op
Bisseling P et al. [21,27]	RHA	3	3	77.3 ± 11.2	NR	NR	NR	NR	5 ± 0.75	7 ± 0.25	57 ± 4	98 ± 0.5	0.1 ± 0.1	1.3 ± 0.175	0.1 ± 0.1	0.9 ± 0.225
	THA	6	3	55.6 ± 11.8	NR	NR	NR	NR	4 ± 1	7 ± 0.5	53 ± 3.75	98 ± 0.5	0.1 ± 0.1	0.9 ± 0.125	0.1 ± 0.1	0.1 ± 0.175
Costa ML et al. [15,22]	RHA	13	1	NR	NR	NR	NR	NR	NR	NR	48.6 ± 14.2	88.4 ± 2.2	NR	NR	NR	NR
	THA	22	3	NR	NR	NR	NR	NR	NR	NR	50.1 ± 13.5	82.3 ± 4.8	NR	NR	NR	NR
Hersnaes PN et al. [24]	RHA	0	6	NR	NR	NR	NR	NR	NR	NR	NR	97.66 ± 5.5	NR	0.92 ± 0.21	NR	1.21 ± 0.53
	THA	2	0	NR	NR	NR	NR	NR	NR	NR	NR	99.3 ± 1.52	NR	1.67 ± 0.36	NR	1.36 ± 0.53
Karampinas PK et al. [16]	RHA	0	NR	NR	NR	NR	72.36 ± 10.16	94.55 ± 3.01	4.07 ± 1.49	8.13 ± 1.14	60.3 ± 39.94	95.6 ± 71.95	NR	NR	NR	NR
	THA	0	NR	NR	NR	NR	65.58 ± 10.89	93.35 ± 34.79	3.5 ± 1.15	6.75 ± 1.13	56.5 ± 11.88	93.7 ± 53.61	NR	NR	NR	NR
Konan S et al. [23,32]	RHA	NR	1	NR	NR	NR	NR	88.61 ± 3.4	NR	6.5 ± 1.9	NR	NR	NR	NR	NR	NR
	THA	NR	7	NR	NR	NR	NR	88 ± 15.7	NR	5.9 ± 1.7	NR	NR	NR	NR	NR	NR
Kostretzis L et al. [20,30]	RHA	2	2	NR	NR	NR	46.5 ± 14.9	85 ± 16	NR	7.2 ± 1.8	NR	NR	NR	1.7 ± 2	NR	1.4 ± 1.1
	THA	3	5	NR	NR	NR	54.31 ± 4.5	94 ± 7.8	NR	6.7 ± 1.8	NR	NR	NR	3.8 ± 3.2	NR	1.9 ± 1
Penny J et al. [25]	RHA	1	1	113 ± 15	625 ± 467	24 ± 2.8	50 ± 21	81 ± 3	5.8 ± 2.2	7.3 ± 1.8	63 ± 10	93 ± 10	NR	NR	NR	NR
	THA	3	0	83 ± 12	753 ± 315	15 ± 2.6	55 ± 16	101 ± 8	6.3 ± 1.8	7 ± 2	56 ± 9	91 ± 14	NR	NR	NR	NR
Tao R et al. [26]	RHA	0	0	98 ± 12	353 ± 79	NR	NR	NR	NR	NR	NR	90.4 ± 2,4	NR	NR	NR	NR
	THA	0	1	79 ± 9	429 ± 109	NR	NR	NR	NR	NR	NR	90.8 ± 5.1	NR	NR	NR	NR
Vendittoli PA et al. [17,18,19,28,29,31]	RHA	21	9	101 ± 18.1	529 ± 262.7	17.2 ± 3.4	52.7 ± 16.2	10.7 ± 16.2	NR	6.3 ± 4.6	NR	NR	0.16 ± 0.16	0.92 ± 0.87	1.02 ± 0.64	2.09 ± 1.93
	THA	21	5	87 ± 24.1	543 ± 467.2	15.1 ± 5	55 ± 18.9	8.81 ± 1.8	NR	6.4 ± 4.6	NR	NR	0.2 ± 0.26	0.76 ± 0.87	1.05 ± 0.82	1.42 ± 0.74

For each series of patients, the author of the most recent paper is reported in the tab.

**Table 3:** Risk of bias assessment using the RoB2 tool.

Study	D1	D2	D3	D4	D5	Overall
Bisseling P et al., 2015 [27]						
Costa ML et al., 2012 [15]						
Costa ML et al., 2018 [22]						
Garbuz DS et al., 2010 [32]						
Hersnaes PN et al., 2021 [24]						
Karampinas PK et al., 2014 [16]						
Konan S et al., 2021 [23]						
Kostretzis L et al., 2021 [30]						
Lavigne M et al., 2010 [20]						
Penny J et al., 2013 [25]						
Rama KR et al., 2009 [17]						
Smolders JM et al., 2011 [21]						
Tao R et al., 2018 [26]						
Vendittoli PA et al., 2006 [31]						
Vendittoli PA et al., 2010 [18]						
Vendittoli PA et al., 2013 [19]						
Vendittoli PA et al., 2020 [29]						
Vendittoli PA et al., 2010 [28]						

Red—“high risk of bias”; yellow—“some concerns”; green—“low risk”.

## Data Availability

No new data were created in this study. Data sharing is not applicable to this article.
